# Evolution and Clinical Advances of Platelet-Rich Fibrin in Musculoskeletal Regeneration

**DOI:** 10.3390/bioengineering10010058

**Published:** 2023-01-03

**Authors:** Ragunanthan Narayanaswamy, Bishnu Prasad Patro, Naveen Jeyaraman, Prakash Gangadaran, Ramya Lakshmi Rajendran, Arulkumar Nallakumarasamy, Madhan Jeyaraman, Prasanna Ramani, Byeong-Cheol Ahn

**Affiliations:** 1 Department of Orthopaedics, Rathimed Speciality Hospital, Chennai 600040, India; 2 Department of Orthopaedics, All India Institute of Medical Sciences, Bhubaneswar 751019, India; 3 Indian Stem Cell Study Group (ISCSG) Association, Lucknow 226010, India; 4 Department of Nuclear Medicine, School of Medicine, Kyungpook National University, Kyungpook National University Hospital, Daegu 41944, Republic of Korea; 5 BK21 FOUR KNU Convergence Educational Program of Biomedical Sciences for Creative Future Talents, Department of Biomedical Science, School of Medicine, Kyungpook National University, Daegu 41944, Republic of Korea; 6 Department of Orthopaedics, ACS Medical College and Hospital, Dr. MGR Educational and Research Institute, Chennai 600056, India; 7 Department of Biotechnology, School of Engineering and Technology, Sharda University, Greater Noida 201310, India; 8 Dhanvanthri Laboratory, Department of Sciences, Amrita School of Physical Sciences, Amrita Vishwa Vidyapeetham, Coimbatore 641112, India; 9 Center of Excellence in Advanced Materials & Green Technologies (CoE–AMGT), Amrita School of Engineering, Amrita Vishwa Vidyapeetham, Coimbatore 641112, India

**Keywords:** platelet-rich fibrin, cytokines, intercellular signaling, growth factors

## Abstract

Over the past few decades, various forms of platelet concentrates have evolved with significant clinical utility. The newer generation products, including leukocyte-platelet-rich fibrin (L-PRF) and advanced platelet-rich fibrin (A-PRF), have shown superior biological properties in musculoskeletal regeneration than the first-generation concentrates, such as platelet-rich plasma (PRP) and plasma rich in growth factors. These newer platelet concentrates have a complete matrix of physiological fibrin that acts as a scaffold with a three-dimensional (3D) architecture. Further, it facilitates intercellular signaling and migration, thereby promoting angiogenic, chondrogenic, and osteogenic activities. A-PRF with higher leukocyte inclusion possesses antimicrobial activity than the first generations. Due to the presence of enormous amounts of growth factors and anti-inflammatory cytokines that are released, A-PRF has the potential to replicate the various physiological and immunological factors of wound healing. In addition, there are more neutrophils, monocytes, and macrophages, all of which secrete essential chemotactic molecules. As a result, both L-PRF and A-PRF are used in the management of musculoskeletal conditions, such as chondral injuries, tendinopathies, tissue regeneration, and other sports-related injuries. In addition to this, its applications have been expanded to include the fields of reconstructive cosmetic surgery, wound healing in diabetic patients, and maxillofacial surgeries.

## 1. Introduction

Platelet concentrates are products that are obtained by centrifuging a blood sample to separate the platelets from the plasma. They concentrate the platelets, fibrin, and leukocytes, so transforming them into a clinically relevant and useful product (depending on the technique that was utilized) [1,2]. Second-generation platelet concentrates (platelet-rich fibrin, PRF) have protocols that are simpler, less expensive, and quicker than those of the first-generation (platelet-rich plasma) [3,4]. In France since 2001, autologous leucocyte- and platelet-rich fibrin (L-PRF) has been utilized clinically as a biomaterial, which requires neither anticoagulant nor bovine thrombin [2,3,5]. Without any additives, platelets are activated within a few minutes after contacting the walls of the tube, which stimulates the coagulation cascade [6,7,8]. The success of PRF isolation depends on the time taken from the speed of blood collection to the transfer of tubes to the centrifugation machine [9,10,11]. Quick handling of PRF isolation is the best way to obtain a clinically and therapeutic usable L-PRF clot [3,8]. 

The cellular composition and 3D organization of PRF are still debated. The cellular composition, histomorphology of the fibers, and cytokine profile of various forms of PRF have been extensively evaluated by various researchers [3]. L-PRF promotes the process of healing and regeneration at the location of the damage by allowing for the controlled release of molecules (vascular endothelial growth factor (VEGF), platelet-derived growth factor (PDGF), transforming growth factor-β (TGF-β), and anti-inflammatory cytokines) over a prolonged period, and also stimulates healing [12]. These factors stimulate neoangiogenesis and the proliferation and differentiation of osteogenic and chondrogenic cells [12]. In the modern era, the usage of newer autologous platelet concentrates is not only confined to dentistry [13,14,15] but also to other diseases such as ulcers and necrosis of skin in the field of plastic and reconstructive surgery [16,17,18,19], and also musculoskeletal lesions [20].

## 2. Evolution of Platelet Concentrates

### 2.1. Fibrin Glue

More than 40 years ago, several compounds produced from blood were used as a channel to accelerate the body’s natural capability to heal on its own. In the beginning, the only time platelet concentrates were utilized was to treat severe cases of thrombopenia to stop bleeding. The upbringing of platelet concentrates to encourage the repair of tissues began with the recognition of adhesive characteristics of the fibrin matrix and the availability of biological growth factors of interest [21]. The production of autologous fibrin glue begins with the utilization of donor plasma, followed by the incorporation of thrombin as well as calcium to kickstart the process of polymerization. The addition of thrombin, as well as calcium, to the processed plasma triggers the polymerization process, generating the bioactive fibrin adhesives of clinical utility, which are similar to the final stages of the coagulation cascade [22]. The major drawback with the application of commercially available fibrin adhesives in regenerative medicine is the possibility of disease transmission (from pooled or single-donor plasma), which can be minimized using autologous plasma [23]. However, the expense of their fabrication, more processing time, the various concentrations, and the tensile strength of fibrin is not adequately determined.

### 2.2. Platelet-Rich Plasma (PRP)

Platelet-rich plasma (PRP) is the first generation of platelet concentrate with the amalgamation of various growth factors and the high qualities of fibrin glue, which in turn results in increasing healing as well as the regeneration potential of tissues [24,25]. Even though PRP preparation and administration protocols are not standardized, it has been extensively employed in regenerative therapies [26]. Till now, more than 40 PRP preparatory techniques from autologous whole blood have been adopted [13]. The major drawbacks of PRP are (1) the quality and therapeutic efficacy of PRP rely on the concentration of platelets in the final platelet concentrate of the individual, (2) the time interval between the isolation to the administration of PRP as activated PRP release 95% growth factors within the first 10 min, and (3) the addition of anticoagulants disrupts the coagulation cascade and activates to form fibrin clots [27,28]. The process of the rapid outburst of growth factors and the lack of homogeneity in the PRP isolation protocols contributed to the development of a novel platelet concentrate with an ability to overcome the constraints that were previously outlined.

### 2.3. Platelet-Rich Fibrin (PRF)

**Figure 1:**
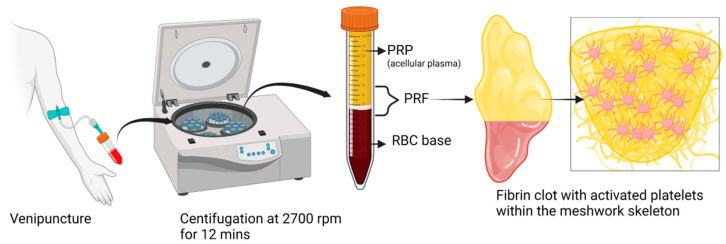
Schematic representation of PRF preparation. Created with BioRender.com.

**Figure 2:**
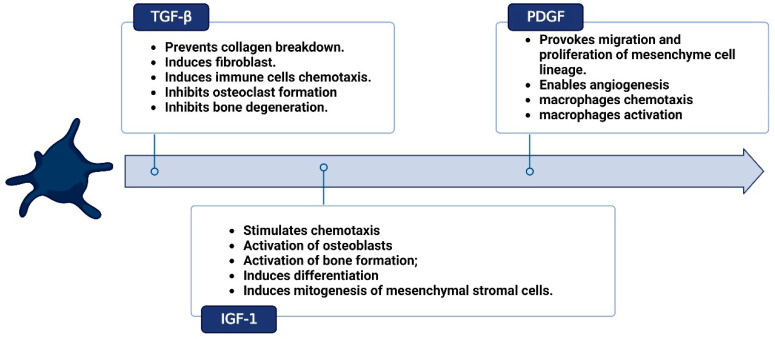

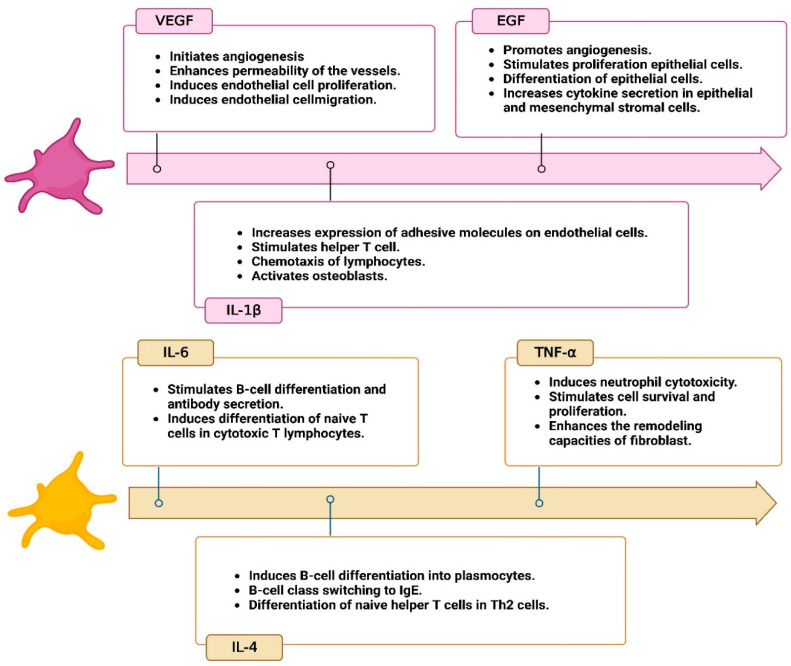
Cytokines profile in platelet-rich fibrin (PRF). Created with BioRender.com.

PRF is a solid membrane of fibrin-containing platelets, leukocytes, and macrophages that represents a big step forward in the field of regenerative medicine, as depicted in Figure 1 [29]. Growth factors present in PRF enhance tissue regeneration, neovasculogenesis, and bacteriostasis, as depicted in Figure 2. PRF is an ideal 3D scaffold for the various stages of the tissue healing process because it is simple to prepare, inexpensive, poses few dangers to patients, and can even be used outside the hospital setting [29,30]. Miron et al. reported a novel technique of harvesting concentrated PRF with a 10-fold increase in platelet and leucocyte counts [31]. Dohan et al. reported no statistically significant difference in the platelet counts and cytokines profile (TGF-β1, PDGF-BB, and IGF-1) in various parts of PRF and supernatant exudate samples [32]. With the presence of leucocytes, Dohan et al. reported PRF as a surgical additive for reducing postoperative infections. They analyzed IL-1β, IL-6, and TNF-α (proinflammatory cytokines), IL-4 (anti-inflammatory cytokine), and VEGF (growth promoter of angiogenesis) in a platelet-poor plasma (PPP) supernatant and a PRF gel. The level of the cytokines and the growth factor correlated positively with that obtained from the PPP and PRF gels. They concluded that PRF gel possesses immunoregulatory properties with retro control inflammation abilities [33]. O’Connell described the safety precautions in the clinical use of PRF using a kit method in terms of system architecture and material hazards in the kits [34].

**Figure 3:**
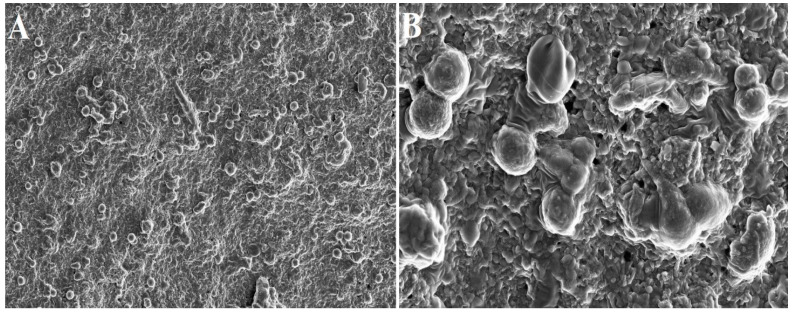
Electron microscopic images of PRF scaffold: (**A**) 2000× and (**B**) 10,000× magnification. The spherical structure indicates inactivated platelets.

In a nutshell, a blood sample is obtained without the use of anticoagulant in tubes made of glass-coated plastic with a capacity of 10 milliliters and is immediately centrifuged between 2700 and 3000 revolutions per minute (about 400 g) for a period of 10 to 12 min [3,35]. When the centrifugation process is complete, the red blood cells (RBCs) will be found at the bottom level of the centrifuge tube, and the platelet-poor plasma will be seen at the top of the tube. In the middle portion of the tube, between the layer of PPP and the layer of RBCs, a PRP clot develops [3,35]. This clot traps a significant number of platelets, leukocytes, cytokines, and growth factors. When the top PPP layer is removed, the PRF that has been obtained can be gathered with relative ease. Allowing platelet activation and fibrin polymerization to occur in the same manner that they would in a normal state is the essential principle. The platelets become immediately activated when it contacts the side wall of the centrifuge tube, which ultimately results in the creation of a thick fibrin network [36]. Because of this, the collection of blood and its subsequent transfer into centrifuge tubes must be completed as quickly as humanly possible, specifically within a maximum of two minutes and thirty seconds. If this interval is extended, fibrin polymerization will occur in a dispersed manner, and the PRF that is created will not be therapeutically useful. The PRF thus obtained does not contain any anticoagulants or any other biochemical alterations that are produced artificially. Altering the methodology for centrifugation of PRF can have an effect not only on the structure but also on the density of PRF clots. Standardization of centrifugation methods is now essential to manage the content of growth factors and fibrin architecture [35,37,38]. Additionally, the impacts of alterations made to the initial manufacturing process need to be clarified to determine how they affect PRF activity. The microdissection of a PRF sample under an electron microscope is depicted in Figure 3.

Kobayashi et al. collated the rate of release of growth factors over time for PRP, PRF, and advanced PRF (A-PRF). PRP demonstrated the rapid distribution of growth factors, whereas PRF and A-PRF are more appropriate for the sustained release of growth factors over a prolonged period [39]. In comparison to conventional PRF, A-PRF was capable of releasing a much greater quantity of growth factors. Qiao et al. quantified five key growth factors such as basic fibroblast growth factor (bFGF), PDGF-BB, TGF-β1, IGF-1, and VEGF using ELISA in activated platelet-rich plasma (PRP) and platelet-rich fibrin (PRF) and found that the concentrations of bFGF in PRF were noticeably greater than those in the activated form of PRP whereas no significant differences were found with the number of other growth factors [40]. Miron et al. concluded that 700 g for 8 min provides a better concentration of platelets, leucocytes, and macrophages in PRF gel subjected to the patient’s hematocrit variability when compared with 24 different PRF preparation protocols [9].

PRF, the second generation of platelet concentrate, was brought forward by Choukroun et al. in 2001 [41], which has several advantages over PRP, which are as follows: (1) As the PRF preparation technique does not involve any additives (anticoagulants, thrombin, or calcium chloride), the cascade of wound healing is preserved and further eliminates the risks involved in the utilization of bovine thrombin. (2) PRF possesses an immunoregulatory and antimicrobial response, which aids in the process of healing wounds. (3) PRF gel develops a thick fibrin meshwork spontaneously, which will allow for a slower degradation rate and provide a pulsatile burst of growth factors constantly at the desired site. (4) PRF gel possesses a high degree of flexibility and elasticity. (5) In addition, the production and standardization of PRF are more reliable and economical when compared with PRP production [8]. The shortcomings of PRF are (1) the success of PRF isolation depends on the speed of blood handling, (2) PRF gel must be used immediately, as it loses structural integrity and modulus of elasticity over some time, and (3) the storage of PRF gel is not possible due to dehydration and potential bacterial contamination [8].

## 3. Other PRF Formulations

### 3.1. Advanced PRF (A-PRF)

**Figure 4:**
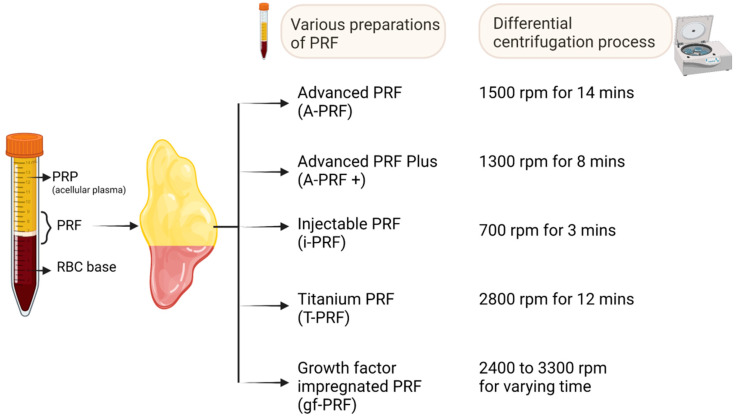
Various forms of PRF preparations. Created with BioRender.com.

Advanced PRF (A-PRF) is generated by increasing the centrifugation time while decreasing the rpm (1500 rpm for 14 min), as depicted in Figure 4, causing leucocytes to shift to the bottom of the tube. The rise in neutrophilic granulocytes at the bottom of the clot facilitates the differentiation of naïve monocytes/macrophages into activated form. When compared with the standard (S-PRF 2700 rpm for 12 min) and leucocyte-rich, platelet-rich fibrin (L-PRF), the resultant A-PRF contains a superior total amount of viable neutrophils, lymphocytes, various growth factors (TGF-β1, VEGF, PDGF, EGF, and IGF-1), and mediators such as osteocalcin, osteonectin, fibrinogen, vitronectin, fibronectin, and thrombospondin [12]. The presence of immune cells and growth factors improves the regenerative potential by enhancing intercellular signaling and tissue-specific macrophage differentiation [12]. The morphology of A-PRF shows a looser structure with more interfibrous space plugged with an enhanced number of cells in the gel. The whole A-PRF clot contains CD61^+^ (platelets) cells, and about two-thirds of the A-PRF gel contains CD15^+^ (neutrophilic granulocytes) cells [12]. With the presence of profound TGF-β1 and PDGF-AB levels in A-PRF gel, the concept of “guided smart tissue engineering” came into existence. However, despite the extensive research that has been conducted, only a small amount of research data is currently available; therefore, new studies are required to evaluate the advantages of A-PRF and L-PRF, as well as their respective limitations.

### 3.2. Advanced PRF plus (A-PRF+)

The A-PRF technique was altered further, which led to the creation of a new formulation called advanced platelet-rich fibrin plus (A-PRF+). Because the amount of force applied during centrifugation has a direct influence on the total number of cells that become entangled within the PRF matrix, researchers shortened the centrifugation force, which decreased the cell count. The mechanical properties of various forms of PRF differ by releasing growth factors at a constant rate [42,43,44]. A-PRF+ gel released higher concentrations of PDGF and TGF-β1 on days 7 and 10 of scaffold implantation, whereas EGF was released within 24 h, and the delayed release of VEGF was observed, as they are plugged within the fibrin and fibrinogen meshwork [8]. 

The concept of the “low-speed centrifugation concept” (LSCC) used in isolation of A-PRF and A-PRF+ creates higher diameter pores in the fibrin meshwork, which allow neoangiogenesis to perfuse the peripheral edges of the scaffold [45]. The fibers of A-PRF+ are thin and elongated, followed by a preferential and oriented direction where platelets, leucocytes, and macrophages are plugged-in [46]. A-PRF+ possesses maximum tensile strength, resistance to traction, and modulus of elasticity compared with A-PRF. The porosity of A-PRF+ appears as porous tangled spaced fibers of high density directed longitudinally and laterally. The morphology appears as thin biofibers with higher polymerization maturity. The biological signature of A-PRF+ is high when compared with A-PRF due to enhanced porosity with strong fibrin architecture, which helps in biological augmentation in the clinical setting [45,47].

Fujioka-Kobayashi et al. developed the A-PRF+ preparation routine, which consists of reducing the centrifugal speed while increasing the time (1300 rpm for 8 min), as depicted in Figure 4 [48]. In comparison with A-PRF and L-PRF, A-PRF+ showed a considerable increase in the number of released growth factors. Further, A-PRF+ showed increased migration and proliferation potential in the cellular medium [8]. This improved outcome may be connected to a higher concentration of neutrophilic granulocytes and lymphocytes entrapped in the fibrin mesh. Additionally, after 3 and 7 days of cultivation, higher amounts of collagen1 mRNA were seen in gingival fibroblasts that had been grown and exposed to A-PRF+ [8]. The results obtained indicate that the regenerative potential in PRF formulations was generated with reduced centrifugation speed and time [45].

### 3.3. Injectable PRF (i-PRF)

One of the primary disadvantages of PRF in comparison to PRP is that it can only be obtained in the form of a gel, which makes it unsuitable for injection. Since PRP may be administered in liquid form, it is versatile enough to be employed by itself or in conjunction with biomaterials across regenerative medicine’s subspecialties. This injectable version of PRF (i-PRF) is synthesized from the blood sample spun at 700 rpm for 3 min in a centrifuge tube (as depicted in Figure 4) without any anticoagulants [9,49,50,51]. Due to the hydrophobic surface of the plastic tubes used in this procedure, the coagulation process is not activated as effectively. This method forms a platelet-rich yellow layer at the top; it can be aspirated with ease and is readily available to be used in an injectable form.

i-PRF possesses a finer pore size of 10 μm, which makes the scaffold plug in more cells and growth factors [52]. Microdissection of i-PRF revealed the adherence of platelets and leucocytes in the pore wall and the edge of the fibrin scaffold. The fibrils appear as sparse fibrous reticular structures [52]. The sustained release of growth factors (PDGF, VEGF, TGF-*β*, IGF, FGF, and EGF) was observed with i-PRF gel over 15 days. i-PRF enhances the proliferation of bone-marrow-derived mesenchymal stromal cells (MSCs) when cocultured with bone marrow. i-PRF downregulates the expression of MMP-1 and -9 and upregulates the expression of TIMP-1 and -2 [52].

i-PRF facilitates re-epithelialization and neovasculogenesis in a full-thickness skin defect mouse model [52]. Since i-PRF is an uncrosslinked liquid scaffold in a conducive form, it secretes high concentrations of growth factors for 2 weeks and accelerates the wound healing process [53]. i-PRF increases collagen type 1 and 3 in wound beds and helps in the deposition of ECM in the skin defect area. i-PRF initiates neovasculogenesis, recruits MSCs, and helps in tissue regeneration [53]. In addition, in comparison to PRP, i-PRF demonstrated higher levels of mRNA expression of PDGF after 72 h, TGF- after a week, and collagen 1 expression within a week [8,49,53]. The findings may suggest that PRF has a more intense biological effect than PRP does; however, this theory needs to be investigated further before being accepted.

### 3.4. Titanium-PRF (T-PRF)

Titanium is biocompatible, hemocompatible, and noncorrosive. Since it displays excellent osseointegration, titanium has become a promising material in orthopedics. Titanium-PRF (T-PRF) is produced by using a sample of blood spun at 2800 rpm for about 12 min, as depicted in Figure 4 [54], which uses titanium tubes made from medical-grade titanium. T-PRF’s fibrin meshwork exhibits more firm, thicker, and more woven consistency and integrity. T-PRF alleviates the issues of cross-contamination with silica and glass tubes [54,55]. Similarly, authors also showed that the fibrin network formed by T-PRF covers a statistically larger area and is thicker than the fibrin network formed by L-PRF. This indicates that T-PRF may be active in the tissues for a longer time than L-PRF [42].

With H and E staining, T-PRF exhibits a highly and densely organized meshwork of fibrin with continuous integrity [56]. In immunofluorescent staining, T-PRF appears more mature and denser than L-PRF. In SEM analysis, the T-PRF clot appears as a well-organized fen-like matrix containing slender fibrin fibrils along with plugged-in platelets [56]. Though it possesses better hemocompatibility, T-PRF clot displays more polymerized fibrin meshwork [54,57]. Ravi et al. reported high tensile strength and modulus of elasticity for T-PRF when compared with L-PRF and A-PRF [42]. In the degradation test, they observed a delayed degradation of T-PRF (82.27%) clot than A-PRF (84.18%) and L-PRF (85.75%) [42].

Preclinical studies reported increased duration and speed of centrifugation help in the recovery of clinically mature T-PRF, which shows superior osseous regeneration than L-PRF. Due to a longer resorption rate and stronger fibrin meshwork, T-PRF displays prolonged release of growth factors. When T-PRF is combined with bone grafts, it offers excellent osteointegration, bony growth, and hemostasis when compared with L-PRF [58].

### 3.5. Growth-Factor-Impregnated PRF (gf-PRF)

Growth-factor-impregnated PRF is a modified form of i-PRF. It is manufactured by centrifuging the blood sample at a varying speed of 2400 to 3300 rpm for a specific period (oscillating tubes) without anticoagulants, as depicted in Figure 4 [9,20]. Finally, the aspirate rich in platelets is separated and used in an injectable form. However, there are restrictions placed on the reference data for the PRF protocol alterations that are named.

## 4. Clinical Applications of PEF in Musculoskeletal Conditions

### 4.1. Cartilage Regeneration

With an inferior intrinsic potential of cartilage regeneration, the augmentation of regenerative medicine products, such as PRP, PRF, MSCs, and MSC-derived EVs, plays a significant role in the management of cartilage disorders [59]. Chien et al. reported the amalgamation of PRF within a biodegradable fibrin scaffold for enhancing the proliferation and differentiation of chondrocytes, enhanced cell growth rate significantly, and upregulated mRNA expression of type-II collagen and GAG synthesis [60]. El Raouf et al. compared iPRF and PRP from rabbit blood and reported that iPRF was found to be superior in regulating chondrogenesis genes and counteracting IL-1β effects in osteoarthritis (OA)-like environment [61]. In full-thickness critical-sized osteochondral defects of rabbits, iPRF filled the defects with osteochondral regeneration. Histological examination revealed hyaline cartilage within 4 weeks postoperatively, which is because iPRF promotes chondrocyte proliferation and mRNA levels of SOX-9, collagen type-2, and aggrecan when compared with PRP or control groups. iPRF, with the low-speed centrifugation concept, poses an improved cartilage regeneration compared with PRP [61]. Wong et al. demonstrated a single-stage culture-free method for repairing articular chondral defects by combining PRF and autologous cartilage transplantation. PRF facilitates the proliferation, migration, and differentiation of chondrocytes [62]. Souza et al. demonstrated the proliferation and differentiation of adipose tissue-derived stem cells when combined with PRF membrane [63].

In a rat osteochondral defect model, Metineren et al. demonstrated the regenerative potential of cartilage with PRF [64]. Histologically, the osteochondral regenerated tissue demonstrated the presence of hyaline cartilage at the end of 1-year follow-up [64]. Wang et al. demonstrated superior results with the combination of PRP and PRF gels along with microfracture through an arthroscope on knee cartilage defects in 28 cases [65]. Kazemi et al. reported both macroscopic and histological significant differences between PRF-treated and PRF-nontreated experimentally induced knee cartilage defects in animal models [66]. Wu et al. demonstrated the histological evidence of hyaline cartilage formation with intra-articular injection of PRF combined with bone-marrow-derived MSCs for surgically induced chondral defects in rabbit femoral condyle [67]. With the latest cutting-edge technologies, researchers have regenerated cartilage with PRF techniques.

### 4.2. Tendon Repair, Augmentation, and Regeneration 

Autologous PRF improves the cellular and biomechanical response in tendon injury and enhances the quality of the repair. Dietrich et al. proved the superior healing effects of Achilles tendon in rat model tendinopathy with autologous PRF than PRP. The PRF-treated group showed a delayed release of growth factors over 14 days when compared with the PRP group. H and E staining analysis showed that the PRF-treated group demonstrated enhanced healing rates at both assessment timelines than the PRP and control groups [68]. Anitua et al. reported that the presence of platelets within fibrin matrices enhances the proliferation of tendon cells significantly in sheep Achilles tendon and exhibits higher synthesis of COL1 and growth factors, such as VEGF and HGF [69]. Visser et al. reported a higher concentration of TGD-β1 elution and enhanced tendon cell proliferation through PRF constructs than whole blood clots in a canine tendon cell in vitro [70].

Beitzel et al. studied the cellular response of MSCs to scaffolds (fresh–frozen rotator cuff tendon allograft, human highly cross-linked collagen membrane, and porcine noncross-linked collagen membrane) in comparison with PRF- and fibrin-matrix-based PRP. They observed a significant number of MSCs adhered to both the noncross-linked porcine collagen scaffold and PRF than the fresh–frozen rotator cuff tendon allograft [71]. Zumstein et al. reported the long-term elution of growth factors from L-PRF in rotator cuff repair. They emphasized (a) the highest concentration of platelets and leucocytes were observed with 400× *g*, (b) sustained release of growth factors, such as TGF-β1, VEGF, and MPO, in the first 7 days of L-PRF clot cultured in the medium, and (c) enhanced growth factor release (CXCL4, IGF-1, PDGF-AB, and VEGF) in the gelatinous group when compared with the dry group, and concluded that the gelatinous type of L-PRF delivers growth factors for up to 28 days and helps in augmenting rotator cuff repair [72].

Castricini et al. reported that PRF augmentation might be beneficial in small, medium, large, and massive rotator cuff tears, given the heterogeneity of PRF preparation protocols available in the market [73]. PRF does not improve the retear rates and postoperative functional outcome scores in cases of full-thickness rotator cuff tears operated arthroscopically. No difference in tendon thickness or size of the tendon footprint thickness was observed with rotator cuff tears [74,75,76,77]. Alviti et al. reported that Achilles tendon repair, along with PRF augmentation, displays a significant functional improvement in motion efficacy than Achilles tendon repair alone [78]. The augmentation of PRF in gluteus medius tendon repair help in improving the subjective outcomes of hip-specific physical functioning than in terms of pain or clinical evidence of tendon retear rates [79]. With the available in vitro, preclinical, and clinical evidence, the role of PRF in tendon augmentation and repair has to be explored in a controlled randomized trial for clinical translation as a therapeutic modality.

### 4.3. Sports and Over-Use Related Injuries

With the increased popularity in the usage of platelet products in sports injuries, it is hypothesized that platelet products accelerate tendon ligamentization, leading to early return to daily activities. Theoretically, PRF possesses graft maturation and hemostatic effects along with analgesic effects in the postoperative period. Beyzadeoglu et al. reported superior graft integration and maturation in the proximal third of PRF-treated autologous hamstring ACL reconstruction when compared with non-PRF-treated grafts in complete ACL tear cases [80]. PRF-treated autologous hamstring grafts display lower MRI signal intensity and less fluid in the graft tunnel interface when compared with controls for the entire graft length [80]. Matsunaga et al. observed 78% of the ultimate failure load of PRF repair tissue at 20 weeks in a bilateral central half-resected patellar tendon in a rabbit model and hence proved that PRF tissue enhances ligament healing [81].

### 4.4. Meniscal Injuries

Meniscal injuries pose a greater challenge in management, as they pose a temporal association between partial or total meniscectomy and the development of OA [82,83]. The need for biological modality for meniscal repair warrants (a) a scaffold for adherence with meniscal tissue, (b) intercellular signals for cellular proliferation and ECM synthesis, and (c) an appropriate number of cells to promote tissue healing. Scanning electron microscopic analysis of PRF demonstrates a honeycomb appearance with plugging-in of platelets along with a fibrin skeleton [84]. PRF scaffolds provide anabolic cytokines to enrich the cells. PRF promotes neoangiogenesis as it possesses low thrombin levels for the migration of fibroblasts and endothelial cells, which could help in meniscal healing [85]. Narayanaswamy et al. reported the usage of iPRF in meniscal repair and augmentation [86]. iPRF application holds better and produces significant functional outcomes in partial meniscectomy. Such iPRF elutes growth factors over 4 weeks, which matches with the healing phase of meniscal tears [86]. Wong et al. demonstrated that rabbit’s PRF augments meniscal repair by facilitating the proliferation and migration of meniscocytes and enhancing ECM synthesis. PRF enhanced the synthesis and deposition of ECM by cultured meniscocytes, which were evaluated both morphologically and histologically [87]. Kurnaz et al. concluded that PRP and PRF matrix augmentation on vertical meniscal tears in a rabbit model resulted in early recovery and enhanced repair of meniscus tissue [88]. The role of PRF in terms of healing and regeneration of meniscus tissue needs to be explored.

## 5. Author’s Perspectives

**Figure 5:**
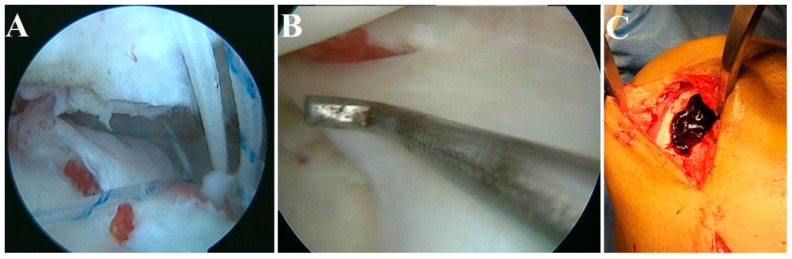
iPRF augmentation in (**A**) rotator cuff tears, (**B**) meniscus repair, and (**C**) chondral defect of the knee.

In our clinical practice, we have utilized iPRF (700 rpm for 3 min) in the augmentation of rotator cuff tears, meniscus repair, and chondral defects of the knee, as depicted in Figure 5. All three cases were followed up for 2 years and showed an excellent functional outcome without any retear or any residual defect in the operated site.

## 6. Limitations

As PRF is a personalized technology and autologous in nature, there is a lack of literature on isolation protocols and standardization in terms of the concentration of growth factors in various PRF. No consensus has been published in terms of the elution of growth factors from PRF gel into the surrounding medium. Regulations on the usage of allogenic and xenogenic PRF must be optimized for the concentration and elution of growth factors as an alternative to autologous PRF, which lacks standardization. Various basic research into all these factors may address the need for clinicians to use PRF in various clinical settings.

## 7. Future Prospects

Various researchers have modified the forms of PRF by impregnating scaffolds or freeze-drying for better mechanical stability and integrity, which are summarized below.

(a) PRF Lysate (PRF-Ly)

The exudate collected after PRF preparation, which is incubated at 37 °C, forms PRF lysate. PRF-Ly contains various cytokines, glycoproteins, and glycans that can initiate neovasculogenesis. PRF-Ly releases an enormous amount of growth factors that initiate the proliferation and differentiation of fibroblasts and synthesize ECM, which can be quantified by ELISA [89]. Dini et al. observed no statistical difference between PRF-Ly and A-PRF in the proliferation of human dental pulp stem cells [90]. Further studies should validate the usage of PRF-Ly in various musculoskeletal disorders for its safety and efficacy.

(b) Lyophilized PRF (Ly-PRF)

Ngah et al. introduced the fabrication of Ly-PRF, which increases the stability of PRF clots. Physically, Ly-PRF appears like a sponge and exhibits a dense, homogeneous interconnected 3D fibrin meshwork with clusters of platelets, leucocytes, and macrophages. Ly-PRF demonstrates relatively rugged and irregular surfaces and a compact texture. Such irregular topography serves as an advantage for the facilitation of osteoblast adhesion and differentiation [29,91]. Morphologically, more cells are situated between the PRF gel and the RBC clot. Such PRF helps in cranial bone repair and regeneration both in vitro and in vivo by providing a pulsatile and continuous release of growth factors. The pore size of Ly-PRF plays an important role in cellular functions, such as cell adhesion, migration, and proliferation. Ly-PRF acts as a reservoir for PDGF-AB, which is a potential growth factor for tissue regeneration [92]. In a rat craniofacial defect model, Ly-PRF provided a maximum defect coverage of about 97% in 6 weeks when compared with L-PRF (84%) [93]. The growth factors released by Ly-PRF enhance the proliferation of bone-marrow-derived MSCs and osteogenic differentiation in vitro [94].

(c) Albumin PRF (Alb-PRF)

Albumin PRF (Alb-PRF) is a byproduct of blood without additives. It involves two processes after centrifugation, namely, heating of the serum and low platelet plasma and incorporation of cells. Such a new biomaterial has been extensively evaluated for its safety and efficacy in tissue regeneration both in vitro and in vivo. Alb-PRF was obtained by centrifuging whole blood at 700 g for 8 min and heating platelet-poor plasma for 10 min at 75 °C [95]. Histological evaluation revealed the complete dispersion of cells in Alb-PRF formulation. The sluggish release of growth factors (TGF-β1 and PDGF-AA/AB) was observed over 10 days in Alb-PRF formulation. Alb-PRF possesses high biocompatibility at 24 h, higher fibroblast proliferation at 5 days, and a substantial rise in TGF mRNA levels and collagen mRNA levels at 1, 3, and 7 days [95]. On denaturing, albumin acquires a 3D structure, leading to the improved stability of the gel [96]. In a preclinical study, Alb-PRF was resorbed for up to 4–6 months when implanted into the subcutaneous area of an animal model. Hence, Alb-PRF acts as a real barrier or biofiller in the defects [97].

## 8. Conclusions

The World Health Organization (WHO) stated that musculoskeletal injuries are the primary reason for morbidity in young individuals worldwide. PRF has overtaken the use of recombinant growth factors, not only for the sake of the treatment of injuries to muscles, tendons, and ligaments but also in the treatment of bone and cartilage injuries. It has gained popularity due to its cost-effectiveness, longer span of life, and more structured delivery to the tissue being targeted. Applying the principles of interventional orthobiologics with PRF reduces the surgical need while treating injuries of the musculoskeletal system, and it augments the success rate of surgical techniques that are being performed nowadays. To optimize PRF isolation and to study the biological features of PRF, newer experimental and clinical research trials are to be conducted in the future. Therefore, the promise of PRF has expanded its role as a therapeutic agent in the regeneration of bone and cartilage when combined with techniques from tissue engineering and grafting.

## Data Availability

Data are contained within the manuscript.
